# Sex ratios at birth vary with environmental harshness but not maternal condition

**DOI:** 10.1038/s41598-019-45316-7

**Published:** 2019-06-21

**Authors:** Ryan Schacht, Douglas Tharp, Ken R. Smith

**Affiliations:** 1 Department of Anthropology, East Carolina University, Greenville, NC 27858 USA; 2 Department of Geography, University of Utah, Salt Lake City, UT 84112 USA; 3 Population Sciences, Huntsman Cancer Institute, University of Utah, Salt Lake City, UT 84112 USA; 4 Department of Family and Consumer Studies, University of Utah, Salt Lake City, UT 84112 USA

**Keywords:** Behavioural ecology, Biological anthropology

## Abstract

The sex ratio at birth (SRB) may be patterned by maternal condition and/or environmental stressors. However, despite decades of research, empirical results from across the social and biological sciences are equivocal on this topic. Using longitudinal individual-level data from a US population during the interwar period (1918–1939), inclusive of three distinct eras (Spanish Flu, Roaring ‘20 s, and the Great Depression), we evaluate predictions from two theoretical frameworks used to study patterning in SRB – (1) ‘frail males’ and (2) adaptive sex-biased investment theory (Trivers-Willard). The first approach centers on greater male susceptibility to exogenous stressors and argues that offspring survival should be expected to differ between ‘good’ and ‘bad’ times. The second approach contends that mothers themselves play a direct role in manipulating offspring SRB, and that those in better condition should invest more in sons. In-line with ‘frail male’ predictions, we find that boys are less likely to be born during the environmentally challenging times of the Spanish Flu and Great Depression. However, we find no evidence that maternal condition is associated with sex ratios at birth, a result inconsistent with the Trivers-Willard hypothesis.

## Introduction

It is well-documented that sex ratios at birth (SRB; presented here as the proportion of boys to girls born at a given point in time) vary within and across species. Understanding this variation has been labeled one of the most elusive concepts in the life sciences today^1^. Over the last several decades a variety of ecological, demographic, economic, and social variables have been evaluated to address this controversy, yet their association with the SRB has been equivocal^2–6^. Here, in an attempt to offer resolution to this conflicted literature, we examine sources of variation in SRB using detailed longitudinal and individual-level data from humans in an early 20^th^ century US population.

There are two theoretical frameworks generally applied to the study of patterning in SRB. The first approach targets greater male susceptibility to exogenous stressors and argues that offspring outcomes should be expected to vary between ‘good’ and ‘bad’ times^4,7,8^. Because males experience higher rates of mortality across nearly all stages of development^9^, likely in part due to their need for greater metabolic investments by mothers to survive^10^, sources of maternal and/or environmental stress are expected to negatively affect males more so than females. Accordingly, we refer to them (and the framework applied here) as ‘frail males’ in response to their predicted greater sensitivity than females to challenging ecological conditions.

A second framework, which does not contest sex differences in exogenous sources of mortality, argues that mothers themselves play a direct role in altering offspring SRB. This theoretical framework, known as Trivers-Willard (TW), makes an evolutionary argument that maternal condition (e.g., social status) ought to play an important role in the amount mothers are willing to invest in sons versus daughters^11^. Specifically, male reproductive success is more variable and this results in greater uncertainty regarding returns to maternal investment. Moreover, because sons are generally more costly to raise to independence (e.g., due to their elevated metabolic needs), this further affects payoffs given maternal condition. Thus, because males with greater access to resources necessary for healthy development and later status competition are expected to be more competitive in the mating market, mothers in good condition are predicted to produce more sons than mothers in poorer condition.

While predictions from the ‘frail male’ and TW frameworks are relatively straightforward, findings in the literature indicate no clear support for either approach. For example, from the ‘frail male’ literature, during the Dutch Hunger Winter (‘Hongerwinter’ in Dutch; 1944–45), where mothers experienced severe resource deprivation over a period of seven months during WWII, SRBs were not significantly different from those in less stressful times^5^. However, other work analyzing data from over three years in China during the Great Leap Forward, known as the Great Chinese Famine (三年大饥荒 in simplified Chinese; 1959–61), finds a precipitous drop in male births during the period of mass starvation^4^ (although alternative findings have been reported^6^). While these results seemingly conflict, one interpretation of differing findings is that shorter periods of deprivation are not enough to trigger male-biased fetal wastage, and instead these events need to be protracted, as in the case of the famine in China.

Results are likewise mixed with respect to the TW literature. While the hypothesis is logically appealing and has been extensively tested in a variety of taxa, the condition of mothers has been found to be both associated and unassociated with sex-biased offspring production across and even within species^12–14^. For example, among Roe deer (*Capreolus capreolus*), mother quality, as evaluated by body weight, has been found to be both positively and negatively associated with SRB^15,16^. Humans too show a pattern of mixed support with women of high socioeconomic status either producing more sons^17^ or displaying no bias^18^.

Accordingly, in an effort to address the ambiguity within the literature, here we approach the question of SRB in three ways. First, we analyze a longitudinal dataset that contains several eras that can be generally categorized as ‘good’ or ‘bad’ in terms of population-level exposure to exogenous stressors such as disease and resource shortage. Importantly, our eras vary in their duration and severity of stress and so allow us to evaluate ‘frail male’ predictions regarding variability in SRB across a heterogeneous fitness landscape. Second, we evaluate maternal condition based on the socioeconomic standing of the mother near the time of a child’s birth (this variable is specifically named in Trivers and Willard’s seminal 1973 paper). Third, following from the approach outlined above, we evaluate how individual mothers navigate time periods of varying environmental quality and the role that context plays on their SRB. Through this three-pronged inquiry, we leverage the insight available from a longitudinal dataset and combine population- and individual-level traits hypothesized to affect SRB. This approach, lacking from previous studies, will allow us to evaluate the role of variation in both environmental harshness and maternal condition on SRB and contribute to the body of work seeking to test these theoretical predictions.

## Methods

Our data were sourced from the Utah Population Database^19^ (UPDB). The UPDB consists of vast genealogical records, originally obtained from the Utah Family History Library, and includes individual-level US Census data as well as birth and death information contributed by both genealogical records and the Utah Department of Health, all spanning the last two centuries. This study has been approved by the University of Utah’s Resource for Genetic and Epidemiologic Research and its Institutional Review Board.

For this study we focus on births between 1918 and 1939 for a total of n = 106,645 (Table 1). The time period under analysis includes three distinct eras that each varied in their length and intensity of environmental stress: the Spanish Flu (1918–20), the Roaring ‘20 s (1925–29), and the Great Depression (1932–36). The Spanish Flu was a global epidemic affecting nearly all regions of the world, Utah included^20^. In fact, in 1919, Utah had the second-highest death rate in the country from the pandemic, with 180 deaths per 100,000 individuals^21^. The disease was largely confined to the winter of 1918–19, but cases continued to be reported through the early part of 1920. In total, an estimated 92,000 individuals were afflicted by the Spanish Flu, thereby affecting some 20% of the population. In response, there was a large public panic resulting in schools being closed, entire towns being placed under quarantine, and the cancellation of all public events. However, after a brief recession in the early 1920’s, from 1925–29 Utah rebounded and grew substantially both economically and in population size, as did much of the US^22^. This era, commonly referred to as the Roaring ‘20 s, was a time period of economic growth and widespread prosperity as both per capita and disposable income rose^23^. This all came to a halt beginning in late 1929, spreading through the early 1930s during the Great Depression, with Utah being among the states hardest hit^24^. Annual per capita income dropped 50% by 1932 and by the spring of 1933, 1/3 of the population was reliant on governmental relief funds due to an unemployment rate hovering around 36% (the fourth highest in the nation).

**Table 1:** Descriptive data summaries for variables and time periods under analysis.

Variable	Total Period (n = 106,645)		Spanish Flu (n = 15,205)		Roaring ‘20 s (n = 21,571)		Great Depression (n = 26,656)	
	Mean	SD	Mean	SD	Mean	SD	Mean	SD
Socioeconomic Status	25.63	10.79	28.95	11.48	26.26	10.48	24.43	10.13
Maternal Age (years)	27.87	6.39	28.19	6.34	28.18	6.45	27.58	6.39
Sex ratio at birth (%)	51.90	0.15	51.12	0.41	53.10	0.34	51.19	0.31

During the time period under study here, we have available Nam-Powers-Boyd Occupation Scores^25^. This is a measure of socioeconomic status (SES; possibly ranging from 1 to 99, but specifically from 3, housekeeper, to 80, financial officer, within our data set) that allows us to explore the role of maternal condition on SRB. A value is assigned based on the household’s SES nearest the year of the child’s birth. We also included the control variables maternal age at birth and child birth year because both are likely associated with SRB. The former is included because it is expected to be negatively associated with the likelihood of having a son due to the greater cost required to bring a son to term, coupled with increasing age-related pregnancy complications^26^. Moreover, controlling for age allows us to more clearly explore the role of SES on SRB following TW predictions because, regardless of age, a mother’s status is expected to affect the relative pay-offs of investment in sons versus daughters. Birth year is included to control for a general secular trend of decreasing mortality rates across time, thereby decreasing fetal, and specifically male, attrition^27^. For our analysis, we attempt to include all women who give birth during the time period under study. However, to avoid the introduction of bias, we exclude farmers because our SES metric does not have variable codes for differing types of individuals involved in farming. Their inclusion would be problematic because farmers are a heterogeneous group and contain, for example, both farm laborers and owners who, respectively, have very different economic standings.

To evaluate predictions from the two theoretical frameworks outlined above, we perform three analyses. For all analyses, we use the statistical program SAS (version 9.4) and logistic regression (PROC LOGISTIC). In our first analysis, we model the relationship of our predictors for ‘bad’ times (Spanish Flu, Great Depression) with whether or not a birth results in a male to evaluate ‘frail male’ expectations. The ‘good’ times era (Roaring ‘20 s) serves as our reference category. Thus, a negative relationship signals that more challenging times are associated with a reduced likelihood of a boy being born. In our second analysis, we again model the probability of a male being born using logistic regression, but now focus on traits of the mother following TW predictions, and include maternal quality as evaluated by SES as a predictor in the model as well as maternal age and child birth year. In our third and final analyses, to test the role of environmental harshness on SRB as mediated by maternal quality, we evaluate the relationship between maternal SES and age and whether or not a son was born in each era.

## Results

**Figure 1:**
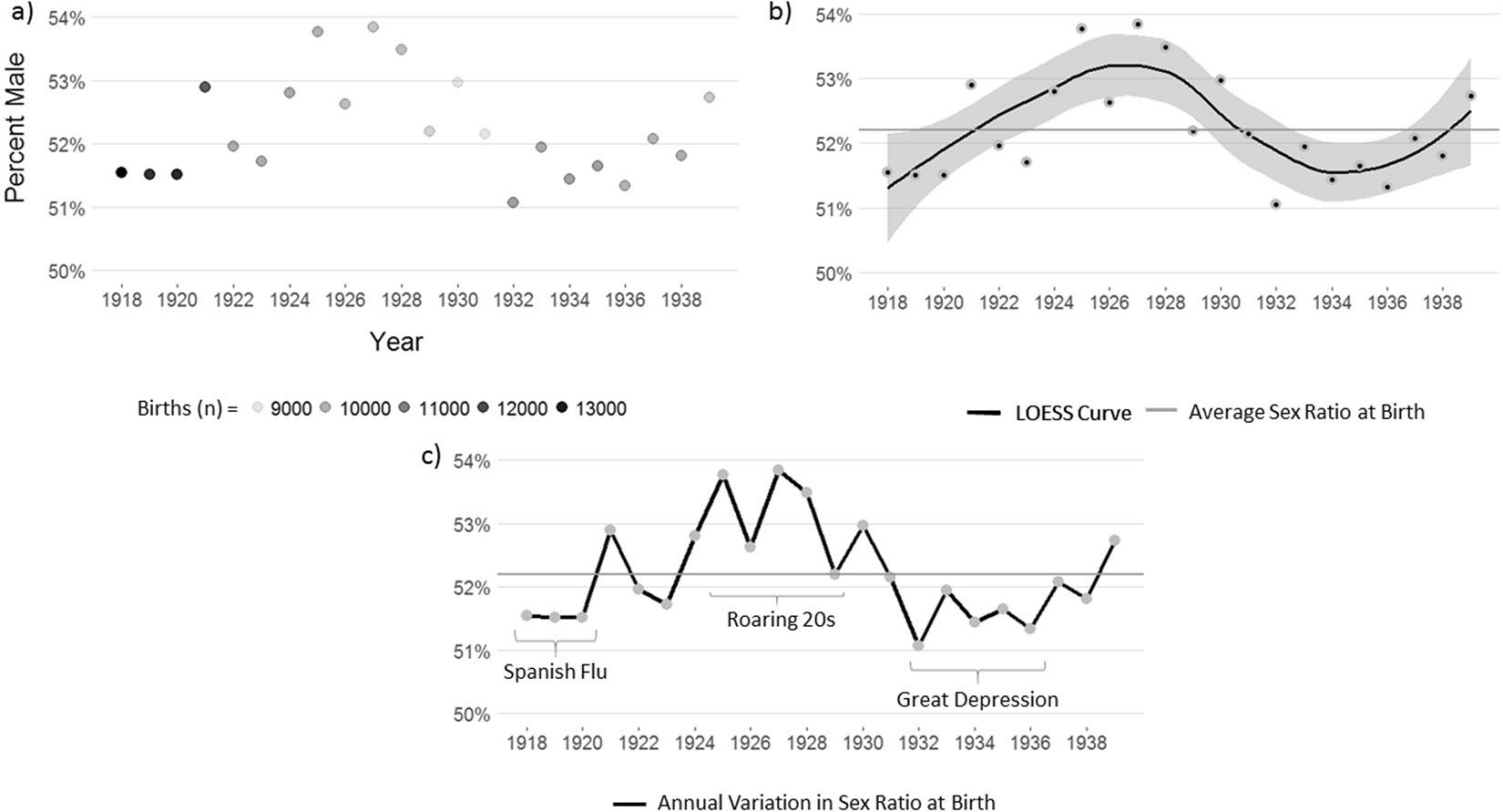
Male percentage of births from 1918–1939 presented in three panels: (**a**) SRB by year plotted with darker circles representing larger birth cohorts. (**b**) LOESS curve fit to the data highlighting nonlinear association over time. (**c**) line graph of interannual variation in SRB with eras outlined.

We present our analysis first by way of a visualization of SRB across time (presented here as the percent of births that are male; Fig. 1). What is easily observable is that more sons than daughters are born from 1918–1939 (Fig. 1a). This result in and of itself is not surprising given that, cross-culturally, generally more boys are born than girls^28^. However, the degree of male bias in the SRB is not uniform and varies interannually from a low of 50.4% male births in 1923 to a high of 54.1% male births in 1925 (mean = 51.9%) and does not appear to be linearly associated with year (Fig. 1b) but instead to be patterned by era (Fig. 1c). During environmentally challenging time periods – Spanish Flu (1918–20) and Great Depression (1932–36) – relatively fewer boys appear to be born than during a time period of rapid economic and population growth and relative abundance of resources – the Roaring ‘20 s (1925–29). Moreover, no trend appears during the inter-era periods. From 1921–24, 1930–1, and 1937–39 annual SRBs shift above and below the average SRB for the time period – something not observed in any one of the eras, which each have uniformly higher or lower SRBs than the average (Fig. 1c).

This visual inspection provides, at least initially, support for ‘frail male’ predictions. To evaluate this relationship statistically, we model the probability of a male being born across ‘bad’ eras (Spanish Flu and Great Depression; n births = 15,205 and 26,656 respectively), with the relatively ‘good’ era (Roaring ‘20 s; n births = 21,571) serving as our reference category. We find that, indeed, relatively fewer boys are born during the challenging environmental times that were likely experienced during the Spanish Flu (b = −0.077, se = 0.022, p < 0.001) and Great Depression (b = −0.084, se = 0.016, p < 0.0001) eras (Table 2).

**Table 2:** Model summary for the relationship between environmental harshness and sex ratio at birth inclusive of our three time periods (n births = 63,432). Time periods Spanish Flu and Great Depression values are compared to the Roaring ‘20 s, while excluding inter-era births.

	Estimate	Standard Error	p-value
Intercept	0.124	0.014	<0.001
Spanish Flu	−0.079	0.021	<0.001
Great Depression	−0.077	0.018	<0.001

We next examine TW predictions and model individual-level traits of mothers across the time period under study. We begin our analysis by exploring whether a son or daughter is born as predicted by mother’s age, SES, and her offspring’s birth year. While SES is predicted to be an important characteristic influencing maternal decisions about sex ratio adjustment, we find no support for that prediction here. Instead, only mother’s age is significant, and negatively associated with SRB (Table 3). That is, older mothers are giving birth to more daughters. To see if this relationship changes in ‘good’ versus ‘bad’ times, we stratified the data by the three eras. We find that during the Spanish Flu and Roaring ‘20 s, mother’s age loses significance (Table 4). That is, maternal traits, as evaluated here, are not significant predictors of SRB. However, during the Great Depression, we recover the negative association between mother’s age and SRB (i.e., older mothers are more likely to produce daughters; Table 4).

**Table 3:** Maternal age and socioeconomic status (SES) as well as offspring birth year predicting sex ratio at birth (n births = 106,645) from 1918–1939.

	Estimate	Standard Error	p-value
Intercept	0.910	1.844	0.622
Age	−0.004	0.001	<0.001
SES	−0.001	0.001	0.086
Birth Year	−0.0004	0.010	0.701

**Table 4:** Maternal age and socioeconomic status (SES) predicting sex ratio at birth by era. *p < 0.05, **p < 0.01, ***p < 0.001.

	Spanish Flu Estimate(SE)	Roaring 20 s Estimate(SE)	Great Depression Estimate(SE)
Intercept	0.1617(0.0813)*	0.1831(0.0663)**	0.2078(0.0581)***
Age	−0.00163(0.00257)	−0.00081(0.00214)	−0.00661(0.00195)***
SES	−0.00245(0.00142)	−0.00138(0.00132)	0.000897(0.00123)

In sum, we find: (1) SRBs are lower (i.e., relatively fewer sons are born) during bad times and higher during good times; (2) no evidence that maternal quality is associated with offspring SRB; (3) older mothers produce more daughters, but only during the prolonged nutritional and economic stress associated with the Great Depression.

## Discussion

Here we evaluate predictions from ‘frail male’ and TW frameworks which each offer explanations for why sex ratios at birth vary within and across populations. We focus our analysis on data from the first half of the 20^th^ century in the US, where the population was experiencing extreme swings in ecological and economic stress. By way of an initial, visual inspection of SRB within the period under study here (Fig. 1), it is clear that a sex-bias in SRB is present and variable across time. This provides an initial confirmation necessary to examine the role of individual and population-level traits on SRB. The novelty of our approach is that we leverage a large, longitudinal dataset that spans eras of varying lengths and levels of ecological harshness to explore how SRB varies, as mediated by maternal quality. Through this approach, we find robust support for ‘frail male’ predictions, yet no support for TW. Accordingly, below we detail our results, situate them within the current literature, and then offer directions for future research, particularly given the long-running debate regarding TW effects.

According to TW, maternal quality is expected to alter the payoff structure for individual mothers regarding their relative investment in sons versus daughters. Because sons are more expensive to produce and their reproductive success is much more variable, mothers in poorer condition are expected to invest more in daughters because their sons will be less competitive in the mating market. However, mothers in better condition (i.e., of a higher status) are able to buffer themselves against the relatively higher costs of producing sons and can make them more competitive through their increased investment potential. While we observe variation in SRB in our sample, we find no evidence that maternal quality as evaluated by SES (using the widely applied Nam-Powers-Boyd measure) is associated with sex-biases in offspring production across time and within each era.

While our findings are consistent with some past work on this topic^18,29^ (i.e., no support for Trivers-Willard), there are other studies that present conflicting results^4,30^. How should we make sense of this? Given our review of the literature, we do not wish to claim that these results are wrong *per se*, but instead that their interpretation of support for TW (or lack thereof) may be problematic. Specifically, a common theme across publications purporting to test TW predictions is that they fail to present data that allow them to do so. For example, Song^4^ looks to the role of nutritional stress on SRB and finds that fewer sons are born during a challenging time period. The author argues that this offers support for TW predictions because mothers in poorer condition are, as expected, having more daughters. However, TW is a not a population-level hypothesis, but instead one that predicts the facultative adjustment of investment in sons versus daughters given a mother’s own condition *relative* to others. Thus, across periods of ‘bad’ versus ‘good’ times, mothers of higher status should still be producing relatively more sons, even if son production is generally depressed across the population. Here, we target this general misinterpretation of theory by specifically assessing the association of maternal quality with SRB across time periods of varying harshness. Through this process, we find no evidence that mothers of higher SES are producing more sons across *any* time period. Thus, here we provide one of the most rigorous examinations of TW to date.

What we do find is clear evidence for the ‘frail male’ hypothesis. Across the time period under study here, the SRB is lower during eras of environmental and economic stress. Specifically, during the Spanish Flu and Great Depression, relatively fewer boys were born than during the much more prosperous Roaring ‘20 s. As we mentioned above, we find no evidence that maternal quality is associated with SRB. However, we find that maternal age is negatively associated with the probability of a son being born. That is, older mothers are producing relatively more daughters, at least when we look across the entire time period. However, when we stratify by era, mother’s age loses significance during the Spanish Flu and Roaring ‘20 s. Why? One interpretation brings to mind the aphorism “a rising tide lifts all boats.” However, during the Spanish Flu, an image of a tide going out is more apropos. This era was one of extreme general stress coupled with a world-wide hysteria that was experienced by all members of the population relatively equally. In Utah, nearly 20% of the population was afflicted by the flu and, of those, approximately 5% died^21^. Accordingly, mothers, generally, were unable to buffer sons from elevated fetal mortality. However, during the Roaring ‘20 s, while there was considerable income inequality, there was a generally experienced prosperity. Most individuals were elevated in economic standing and so maternal traits became unassociated with biases in fetal mortality. However, we find maternal age to be significantly and negatively associated with SRB during the Great Depression (which appears to drive the association observed across time periods; Table 3). This was an era of prolonged nutritional and economic stress, yet the effects were not as acute for all. Accordingly, variation in maternal robustness, as measured by maternal age, resulted in younger mothers being better able to bring a son to term.

One important yet understudied topic is the process by which mothers might manipulate offspring sex in utero^31,32^. Currently, however, we lack clarity regarding a mechanism for sex ratio adjustment, which impedes our ability to interpret results from across animal taxa. Researchers have focused on physiological pathways that might influence SRB near the time of conception. Rather than later in the gestational window, sex-selection operating at this time period is argued to minimize reproductive costs compared to a later curtailment of investment. As a result of this line of inquiry, the role of glucose in determining sex ratios has emerged as a frequently observed phenomenon^33^. From bovines^34^ to mice^35^, higher circulating glucose levels result in relatively more males being produced because, as blastocysts, females survive better in low glucose environments, and males in high. In humans, both high energy maternal diets^36^ and gestational diabetes^37^, which both increase the levels of circulating glucose, have been shown to be associated with a higher likelihood of producing sons. However, this straightforward association is muddied because one of the physiological consequences of maternal stress, which has been shown to result in fewer sons being born, also results in elevated levels of glucose through the increased production of glucocorticoids^38^. To further complicate the role of glucose on SRBs, while we find, as do others^39^, that the likelihood of a son being born decreases with maternal age, maternal glucose levels actually increase with age^40^. This confusing pattern in the literature signals that the mechanism underlying fetal sex manipulation remains understudied and that diet, stress, and age (among other environmental and maternal traits) all interact in ways yet to be fully understood.

Moreover, our findings raise social justice concerns regarding both long-running and contemporary inequalities in exposure to exogenous stressors. It is well-established that individuals of lower SES are more likely to be exposed environmental toxins^41^. Following our results, this may result in SRBs to superficially appear to be patterned by social status, yet be causally-linked to, for example, pollution exposure varying by housing affordability, occupation, and aspects of the built environment. Specifically, dioxin, pesticide, and lead exposure have all been shown to result in fewer boys being born^42,43^. While differences across ethnic groups in SRBs have been noted^44^, and in some cases argued to be biologically-based, these patterns could emerge in response to multigenerational exposure to adverse environmental factors that result in male fetal loss being higher in some groups (those of lower SES) than others. Thus, we argue that false positives in support of TW are likely to exist in the literature, given that SES and exposure to contaminants (which results in fewer boys being born) are negatively associated. While we find no effect of SES, this may be driven by low levels of inequality in environmental exposure within the time period under study here – something in strong contrast across Utah and many other locales today (e.g., SES gradients in air pollution exposure are now well-documented^45,46^).

The Trivers-Willard hypothesis is a long-standing and well-studied evolutionary framework whose predictions have been applied to datasets spanning the social and biological sciences. Despite decades of work, support for TW is equivocal. While the framework itself is logically appealing, the mechanism by which mothers might manipulate fetal sex ratios is largely unknown and so we are left with the question as to what should be more surprising; not reporting a TW effect, or instead seeming to observe one? A likely fruitful direction for future research is to assess TW predictions post-birth^12^. For example, mothers in good condition may not be selectively investing in male fetuses but could instead be biasing their parental care towards sons, resulting in lower mortality rates among these males. The literature on this topic is mixed in terms of results yet could possibly benefit from the approach we apply here.

In sum, to move the literature forward, future researchers need to ensure that they have the data available to them to assess TW predictions. This seems like an obvious statement, but many publications claiming to test TW do not have individual-traits of mothers that may serve as indicators of relative differences in status. While clarity regarding maternal ability to bias the sex ratio is lacking, consensus is growing that mothers produce fewer sons in response to stressful events. In sum, here we find robust support for ‘frail male’ predictions and would like to offer that while adaptive arguments are often appealing, at times simpler explanations may be most appropriate.
